# OCT Angiography Fractal Analysis of Choroidal Neovessels Secondary to Central Serous Chorioretinopathy, in a Caucasian Cohort

**DOI:** 10.3390/jcm11051443

**Published:** 2022-03-06

**Authors:** Rita Serra, Antonio Pinna, Francine Behar-Cohen, Florence Coscas

**Affiliations:** 1 Department of Biomedical Sciences, University of Sassari, 07100 Sassari, Italy; 2 Istituto di Ricerca Genetica e Biomedica (IRGB), CNR, Cittadella Universitaria di Cagliari, 09042 Monserrato, Italy; 3 Centre Ophtalmologique de l’Odeon, 113 Bd Saint Germain, 75006 Paris, France; 4 Department of Medical, Surgical and Experimental Sciences, Ophthalmology Unit, University of Sassari, 07100 Sassari, Italy; apinna@uniss.it; 5 Assistance Publique-Hôpitaux de Paris, Department of Ophthalmology, Ophtalmopole, Hôpital Cochin, 75014 Paris, France; francine.behar@gmail.com

**Keywords:** central serous chorioretinopathy, fractal analysis, indocyanine green angiography, optical coherence tomography angiography, polypoidal choroidal vasculopathy, type 1 choroidal neovascularization

## Abstract

Central serous chorioretinopathy (CSCR) can be complicated by different types of choroidal neovascularization (CNV). The purpose of this study was to investigate the incidence and quantitative optical coherence tomography angiography (OCT-A) features of CSCR-related CNVs. Methods: This was a retrospective multicenter study including 102 eyes of 102 Caucasian patients with acute or complex CSCR. All patients underwent a comprehensive ophthalmological examination. Quantitative OCT-A parameters, including vascular perfusion density (VPD), fractal dimension (FD), and lacunarity (LAC), were measured in CNV eyes. Results: Forty eyes (39.2%) had acute CSCR, whereas the remaining sixty-two (60.8%) had complex CSCR. CNV was observed in 37 (36.27%) eyes, all of which had the complex form. CNVs were classified as type 1 CNV in 11/37 (29.73%) cases and as polypoidal choroidal vasculopathy (PCV) in the remaining 26/37 (70.27%). Overall, the mean VPD, FD, and LAC of CSCR-related CNVs were 0.52 ± 0.20%, 1.44 ± 0.12, and 2.40 ± 1.1, respectively. No significant difference between type 1 CNV and PCV was found. Conclusion: Complex CSCR is often complicated by type 1 CNV and PCV with similar neovascular architecture and branching complexity, a finding supporting the idea that they might be different stages of the same neovascular process. Future OCT-A fractal analysis-based studies that also include other relevant parameters, such as demographics, presentation, morphology on multimodal imaging, and response to treatment, are necessary before drawing any definitive conclusions.

## 1. Introduction

The pachychoroid spectrum includes a group of macular disorders (pachychoroid pigment epitheliopathy, pachychoroid neovasculopathy, polypoidal choroidal vasculopathy (PCV), central serous chorioretinopathy (CSCR), and choroidal excavations), presenting a thick choroid and dilated veins facing areas of choriocapillaris attenuation (“pachyvessels”) [1,2]. Choroidal neovascularization (CNV) is frequently observed in pachychoroid-associated diseases, but whether pathogenic events are similar in CSCR-related type 1 CNV and PCV is unknown.

CSCR is characterized by choroidal vascular abnormalities with subsequent episodes of serous retinal detachment at the posterior pole, typically affecting young and middle-aged adults [3]. Although the underlying pathophysiological mechanism is still not entirely clear, some risk factors, such as psychosocial stress, uncontrolled systemic hypertension, extraocular glucocorticoids, and pregnancy, have been reported [4]. Generally, the natural history of CSCR has a self-limiting course with a good visual outcome; however, a subset of patients show subretinal fluid (SRF) lasting over six months and/or multifocal sites of epitheliopathy, which predisposes to recurrence, CNV formation, and poor long-term visual outcome [3,5,6]. Recently, the later forms of the disease have been identified as “complex” CSCR [6].

CSCR may be complicated by different types of CNV [2,7]. Type 1 CNV extends below the retinal pigment epithelium (RPE), along the roof of flat irregular pigment epithelium detachments (PEDs) on spectral-domain optical coherence tomography (SD-OCT), and inconsistently shows indistinct late leakage on fluorescein angiography (FA) [2,8,9]. Indocyanine green angiography (ICG-A), thanks to its ability to image the choroidal circulation, has shown that many CSCR-related CNVs initially labeled as type 1 CNVs are instead PCVs [10,11]. Therefore, despite being more time-consuming than FA, ICG-A is currently the gold standard for studying the choroidal vasculature and distinguishing type 1 CNV from PCV [12]. Originally, type 1 CNVs and PCVs were considered two specific idiopathic entities in terms of epidemiology, natural history, prognosis, and traditional multimodal imaging [13,14]. Recently, the Consensus on Neovascular Age-Related Macular Degeneration (AMD) Nomenclature Study Group included PCV in the type 1 CNV group [15]. However, PCV and type 1 CNV may show different prognoses and responses to treatment, and whether or not they are unique clinical entities is still a matter of debate.

In the last years, OCT angiography (OCT-A), a novel non-invasive imaging technique, has become more and more popular in clinical practice. OCT-A allows for the detailed evaluation of CNVs secondary to several retinal diseases [16,17] and is particularly sensitive in the detection of angiographically silent CNVs in CSCR [2,18,19]. Furthermore, OCT-A fractal analysis may be helpful in distinguishing neovascular lesions with different natural histories and prognoses, as reported by Serra et al. [16] in a recent study comparing type 1 CNV in the remission phase versus treatment-naïve quiescent CNV in AMD patients. Similarly, Al Sheik et al. [20] demonstrated that OCT-A fractal analysis is useful for differentiating active type 1 CNV from CNV in the remission phase.

To the best of our knowledge, no study has performed OCT-A fractal analysis of type 1 CNVs and PCVs secondary to CSCR. In this context, the purpose of our investigation was to evaluate the incidence and quantitative OCT-A features of CSCR-related CNVs. Potential biomarkers associated with type 1 CNV and PCV, such as vascular perfusion density (VPD), fractal dimension (FD), and lacunarity (LAC), were analyzed.

## 2. Materials and Methods

This was a retrospective review of CSCR cases from two high-volume referral centers (Odeon Ophthalmology Center, Paris, France, and Ophthalmology Unit, Department of Medical, Surgical, and Experimental Sciences, University of Sassari, Sassari, Italy) between March 2018 and July 2019.

The current study was conducted in compliance with the tenets of the Declaration of Helsinki for research involving human subjects. All participants provided informed consent to participate in the present survey.

The inclusion criteria were a diagnosis of CSCR in Caucasian patients and high-quality retinal images. Acute CSCR was defined based on the detection of acute serous retinal detachments that involved the posterior pole, were unrelated to other retinal disorders i.e., dome-shaped macula or staphyloma, uveal effusion syndrome, inherited retinal diseases, acquired vitelliform lesions, tumors, inflammation (Vogt–Koyanagi–Harada disease or posterior scleritis), drug toxicity, tractional maculopathy, retinal vascular diseases, optic pit maculopathy, choroidal nevus, CNV due to other causes, or acute hypertensive retinopathy, and were associated with choroidal hyperpermeability and pachyvessels (focal or diffuse dilation of choroidal vessels in Haller’s layer) [2,6,21]. Complex CSCR was defined as a condition of documented clinical CSCR features (i.e., SRF, RPE changes in the macular area on FA/ICG-A, and SD-OCT) for a duration of at least six months and/or multifocal epitheliopathy diagnosed on mid-phase ICG-A [6].

Exclusion criteria were the presence of any other concomitant ocular disease potentially affecting imaging interpretation (e.g., myopia > 6 diopters), ocular inflammation, angioid streaks, and relevant opacities of the optic media. Furthermore, eyes with poor-quality images on OCT-A (i.e., presence of artifacts secondary to eye movement or poor fixation) were also excluded.

All eligible patients underwent a complete ophthalmic examination. For each patient, all of the following were performed on the same day: best-corrected visual acuity (BCVA) measurement with Early Treatment Diabetic Retinopathy Study (ETDRS) charts, slit-lamp biomicroscopy with dilated indirect fundoscopy, multimodal retinal imaging, including fundus autofluorescence (FA) and ICG-A (Heidelberg Spectralis HRA + OCT, Heidelberg Engineering, Heidelberg, Germany), SD-OCT analysis (Spectralis Domain OCT, Heidelberg Engineering, Heidelberg, Germany) by the acquisition of a macular volume scan (49 B-scans within a 30° × 20° area) centered on the fovea, and OCT-A (AngioVue XRTVue Avanti, Optovue, Fremont, CA, USA), Triton swept-source OCTA (Topcon, Tokyo, Japan), or Spectralis OCT-A (Spectralis; Heidelberg Engineering, Heidelberg, Germany).

After meticulous review of patients’ records and multimodal retinal images, two masked retinal specialists (R.S. and F.C.) classified CSCR as acute or complex. We also assessed the presence of CNVs and determined their subtype according to previously described criteria for multimodal imaging evaluation. On early and intermediate ICG-A frames, type 1 CNV appeared as a hypercyanescent neovascular network, which became a plaque on late frames. PCV was characterized by a typical branching vascular network with aneurysmal dilations on early and intermediate ICG-A frames [17,22,23].

Rare disagreements over the presence or classification of CNVs in images were resolved by open adjudication between readers.

In eyes showing CSCR-related CNVs, the OCT-A angiocube was centered on the CNV, and its appearance and location were compared with ICG-A images. The automated segmentation provided by the OCT-A software was carefully adjusted for correct visualization of the capillary plexus, outer retinal layers, and choriocapillaris to better identify blood flow abnormalities suggestive of CNV and remove segmentation artifacts. Specifically, to avoid differences related to different slab positions, slabs extending from the outer boundary of the outer plexiform layer up to 8 mm beneath the Bruch’s membrane were used to visualize all CNV lesions. To improve CNV visualization, the thickness between two automated segmentation lines was altered, moved through the en face layer of the OCT-A, and then correlated with the cross-sectional OCT findings on the OCT-A platform.

Two investigators (R.S. and A.P.) independently reviewed all OCT-A scans to ensure correct segmentation and image quality for post hoc analysis.

**Figure 1:**
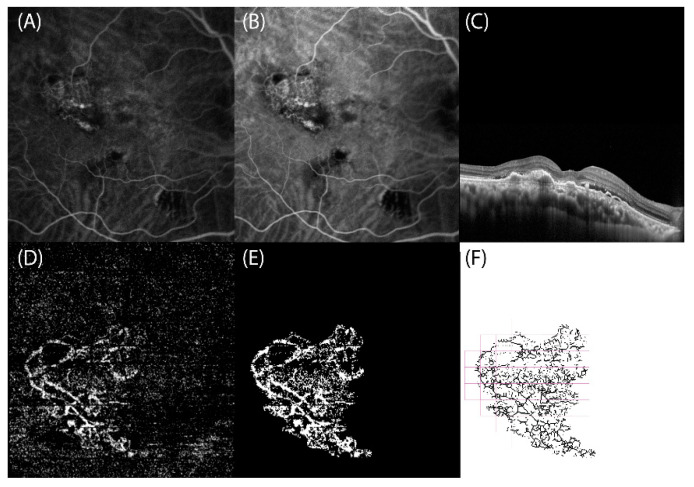
Right eye with polypoidal choroidal vasculopathy (PCV) secondary to complex central serous chorioretinopathy: multimodal retinal imaging and optical coherence tomography angiography (OCT-A) fractal analysis. (**A**,**B**) Indocyanine green angiography frames reveal the presence of a hypercyanescent network corresponding to the branching vascular network (BVN) associated with roundish hypercyanescent structures (polyps) at the terminal ends. (**C**) SD-OCT scan highlights the thick choroid and the subretinal fluid associated with flat irregular pigment epithelium detachments corresponding to the PCV. (**D**) OCT-A slab confirms the presence of a hyperreflective BVN ending with polyps. (**E**) Binarized and (**F**) skeletonized OCT-A image of PCV, obtained using a graphical interface to estimate quantitative fractal parameters.

**Figure 2:**
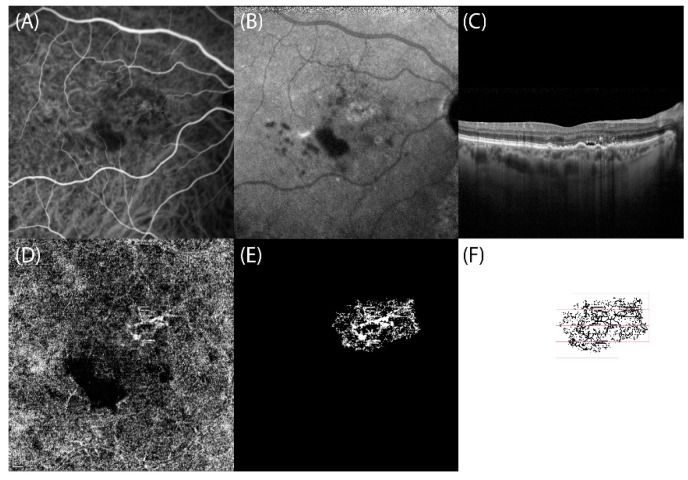
Right eye of type 1 choroidal neovascularization (CNV) secondary to complex central serous chorioretinopathy: multimodal retinal imaging and optical coherence tomography angiography (OCT-A) fractal analysis. (**A**,**B**) Indocyanine green angiography frames reveal the presence of a hypercyanescent network corresponding to type 1 CNV. (**C**) SD-OCT scan highlights the thick choroid and the subretinal fluid associated with flat irregular pigment epithelium detachments corresponding to type 1 CNV. (**D**) OCT-A slab confirms the presence of type 1 CNV appearing as hyperreflective network in the macula region. (**E**) Binarized and (**F**) skeletonized OCT-A image of type 1 CNV, obtained using a graphical interface to estimate quantitative fractal parameters.

To estimate VPD, FD, and LAC, OCT-A slabs showing CNVs were exported in Tagged Image File Format (TIFF) to a previously validated custom graphical user interface built in MATLAB (v.r 2018) for fractal analysis. Images were binarized using the Otsu method [24], and the filtering of speckle noise was achieved by using a median filter with a radius of 2 pixels (small, nonconnected pixels < 10 pixels were removed). Then, the density map was computed, the highest density zone was identified, and the CNV area was calculated by setting a pixel-mm scale. Quantitative OCT-A analysis of VPD, FD, and LAC was performed using a graphical interface. The box-counting method at multiple origins was applied to the image of the binary skeleton to estimate the FD and LAC of the vascular network, which are global indices of morphological complexity and structural non-uniformity, respectively. VPD was defined as the total area of perfused vasculature (on the binarized image) per unit area in a region of measurement (Figure 1 and Figure 2) [17].

The results of descriptive analysis are expressed as numbers and percentages for categorical variables and as means ± standard deviation (SD) for quantitative variables. After testing the data distribution for normality, *t*-test was used, as appropriate. The correlation between FD values and the area of the lesions was evaluated using Pearson’s correlation test. A *p* value < 0.05 was considered statistically significant. The study data were analyzed using the Statistical Package for Social Sciences version 20.0 for Mac (IBM, Chicago, IL, USA).

## 3. Results

A total of 102 eyes of 102 Caucasian patients (72 men, 30 women; mean age: 60.23 ± 13.19 years) with CSCR were included in the study. Among patients, 40 (39.2%) patients (32 men, 8 women) had acute CSCR, whereas the remaining 62 (40 men, 22 women) had complex CSCR. Acute CSCR patients were significantly younger than those with the complex form (mean age: 52.20 ± 9.52 years vs. 68.27 ± 10 years; *p* < 0.0001). Mean BCVA was significantly higher in acute CSCR than in the complex form (87.06 ± 18.58 vs. 79.61 ± 23.32 ETDRS letters; *p* = 0.03)

All demographic and clinical data are summarized in Table 1.

CNVs were identified in 37/102 (36.27%) eyes, all with complex CSCR. They were classified as type 1 CNV in 11/37 (29.73%) cases and as PCV in the remaining 26 (70.27%). The mean age was 65.18 ± 17.35 years in the type 1 CNV group (7 men, 4 women) and 71.50 ± 7.79 years in the PCV group (17 men, 9 women) (*p* > 0.05). The mean BCVA was 68 ± 39.46 ETDRS letters in the type 1 CNV group and 80.5 ± 15.17 ETDRS letters in the PCV group (*p* > 0.05). Both PCN and type 1 CNV eyes had previously been treated with intravitreal injections of anti-vascular endothelial growth factor (VEGF) agents (4.14 ± 2.6 vs. 6.42 ± 4.07 injections, respectively; *p* > 0.05). On SD-OCT, SRF was observed in 2/11 (18.18%) type 1 CNV eyes and in 4/26 (15.38%) PCV eyes.

Comparison of mid/late ICG-A frames and OCT-A slabs revealed a perfect correspondence in shape and location between the hypercyanescent neovascular networks of type 1 CNV and PCV and the hyperreflective network on OCT-A, appearing as a hyperreflective PED on simultaneous structural OCT. Furthermore, in 20/26 (76.9%) PCV eyes, polypoidal lesions appearing as aneurysmatic hypercyanescent dilations on ICG-A corresponded to roundish hyporeflective structures on OCT-A.

All OCT-A findings were matched with the results of multimodal imaging to ensure that the CNV location was correctly identified during segmentation analysis.

Overall, in CSCR eyes with neovascular complications, the mean CNV area, VPD, FD, and LAC were 2.33 ± 2.06 mm^2^, 0.52 ± 0.20%, 1.44 ± 0.12, and 2.40 ± 1.1, respectively. No statistically significant difference was found in terms of area, VPD, FD, and LAC between type 1 CNV and PCV eyes (2.28 ± 1.93 vs. 2.35 ± 2.16 mm^2^, 0.53 ± 0.23% vs. 0.52 ± 0.19%, 1.46 ± 0.15 vs. 1.43 ± 0.10, and 2.10 ± 0.49 vs. 2.53 ± 1.26, respectively). Furthermore, no statistically significant association was found between FD values and neovascular lesion size (r = −0.056, *p* = 0.79). All quantitative OCT-A data are summarized in Table 2.

## 4. Discussion

In the last decade, the use of a multimodal imaging approach has resulted in an increasing incidence of CSCR-related CNV lesions, with reported rates ranging from 20% to 58% [2,25].

Similarly, we found a CNV in 37 (36.27%) out of 102 CSCR eyes, all of which had the complex form. Overall, a CNV was identified in 59.6% of eyes with complex CSCR. This high rate can be explained by the use of multimodal imaging in the diagnosis of CNV. The significant correlation between CNV lesions and complex CSCR is likely to depend on the associated epitheliopathy, as suggested by numerous reports identifying epitheliopathy and long-lasting SRF as important predisposing factors for CNV development [2,7,26]. Epitheliopathy, underlying choroidal abnormalities, and choroidal blood flow deregulation seem to play critical roles [25,27,28]. Vascular remodeling in the context of venous overload may also predispose to CNV occurrence [28]. Other authors have postulated that pachyvessels develop when there is increased choroidal perfusion, which promotes oxidative stress, Bruch’s membrane injury, and diffuse RPE changes, usually seen above mid-phase hyperpermeability plaques [29]. In eyes with long-lasting CSCR, the CNV is frequently silent for several years and is only diagnosed when OCT-A is performed [18,19].

In CSCR, the prevalent pattern is type 1 CNV [26], although type 2 CNV extending through Bruch’s membrane towards the subretinal space has also been described [7]. In this regard, Lee et al. [7] recently described a surprisingly high incidence (76.7%) of type 2 CNV. This discrepancy may be due to the different retinal imaging approaches used to classify CSCR-related CNVs. Alternatively, as previously demonstrated by Chhablani et al. [30], the proportion of type 1 and 2 CNVs may vary, depending on whether or not PCVs are included.

In our study, no type 2 CNV was found. Of the 37/102 (36.27%) eyes with CNVs, 11 (29.73%) had type 1 CNV, and 26 (70.27%) had PCV. Although PCV is relatively uncommon in eyes with wet AMD, with incidence rates ranging from 4% to 9.8% [31,32], in our survey, almost two-thirds of CNVs were PCVs, suggesting a strong correlation between complex CSCR and PCV development. This correlates well with the high incidence of PCV in the Asiatic population, which seems to be unaffected by choroidal thinning with aging [33]. This finding suggests that PCV, which is an aneurysmal dilation of the neovascular process, may also result from choroidal vascular congestion. Another possible explanation for the high rate of PCV in complex CSCR is the long evolution period of silent CNVs, because type 1 CNVs may acquire aneurysmal dilations over time [13,28]. In our Caucasian cohort, PCV patients were approximately 10 years older than those with type 1 CNVs; therefore, we cannot exclude that the former had had CNVs for many years before multimodal retinal imaging could visualize them. 

Recently, several reports have highlighted the efficacy of OCT-A in detecting different CNV types associated with AMD. Indeed, OCT-A may provide detailed images of the different retinal layers, thus allowing the identification of morphological characteristics typical of each CNV type [16,17,21]. Furthermore, Coscas et al. demonstrated that morphological CNV features on OCT-A, such as the presence of a branching vascular network, tiny vessels, and peripheral arcade, are related to the activity status of the CNV [34].

Although quantitative OCT-A parameters are useful biomarkers to objectively distinguish AMD-related CNVs with different natural histories and prognoses [16], we failed to find any statistical difference in terms of VPD, FD, and LAC between type 1 CNV and PCV in CSCR eyes. This result might imply that type 1 CNV and PCV in CSCR eyes share similar neovascular architecture and complexity, thus supporting the idea that they are two stages of the same neovascular process, as suggested by Siedlecki et al. [35] and Hua et al. [36]. 

Our study has several limitations, mainly due to its retrospective nature and the relatively small number of eyes analyzed. 

It is also important to acknowledge that the presence of serous PEDs, SRF, and RPE changes, all typical CSCR features, might have somewhat influenced fractal analysis results. Furthermore, we cannot exclude that fractal analysis results may have been affected by the intrinsic limitations of OCT-A instruments. Indeed, the ability of OCT-A to visualize blood flow is limited to a certain range of flow velocities (minimum, 0.5–2 mm/s; saturation, 9 mm/s estimated for current devices) [37]. Therefore, it is theoretically possible that certain neovascular networks, or parts of them, are not detected by OCT-A, because their flow speed is below the instrument detection limit [17]. Evidence indicates that PCVs can show different flow speeds within the same lesion, which may result in a turbulent flow with minimal or no signal on OCT-A. In fact, PCV consists of a branching vascular network, generally appearing on OCT-A as a hyperreflective network terminating with hyporeflective aneurysmal dilations [38]. 

Finally, we also acknowledge that OCT-A angiocubes provided with the different devices used in this study are characterized by a different number of B-scans, which may lead to a different representation of CNV lesions. However, Munk et al. revealed that no significant differences exist in terms of CNV detection and VD value computation among different OCT-A modules if the correct OCT-A segmentation is used [39]. A similar result was reported by Mastropasqua et al. [40], who failed to find any statistical difference among CNV measurements obtained by different OCT-A devices [40]. By contrast, other authors have reported statistically significant differences between OCT-A devices in terms of CNV area computations, but they argued that such differences may depend on projection artifacts [41].

In summary, our results show that complex CSCR is often complicated by the occurrence of type 1 CNV and PCV with similar neovascular architecture and branching complexity, a finding supporting the idea that they might be different stages of the same neovascular process. However, future studies based on OCT-A fractal analysis but also including other relevant parameters, such as demographics, presentation, morphology on multimodal imaging, and response to treatment, are necessary before drawing any definitive conclusions on whether PCV in CSCR eyes is a specific clinical entity or a variant of type 1 CNV. 

## Figures and Tables

**Table 1:** Demographic and clinical features of central serous chorioretinopathy patients (*n* = 102).

	Acute CSCR	Complex CSCR	*p* Value
Total eyes, *n* (%)	40 (39.21%)	62 (60.79%)	-
Sex			
- Male, *n* (%)	32 (80%)	40 (64.51%)	-
- Female, *n* (%)	8 (20%)	22 (35.49%)	-
Age, mean ± SD (years)	52.20 ± 9.52	68.27 ± 10	<0.0001
BCVA, mean ± SD (ETDRS letters)	87.06 ± 18.58	79.61 ± 23.32	0.03

Categorical variables are presented as *n* (%). Continuous variables are presented as mean ± standard deviation (SD). CSCR = central serous chorioretinopathy. BCVA = best-corrected visual acuity.

**Table 2:** Quantitative optical coherence tomography angiography (OCT-A) parameters of type 1 choroidal neovascularization (CNV) and polypoidal choroidal vasculopathy (PCV) in complex central serous chorioretinopathy eyes.

	Type 1 CNV	PCV	*p* Value
VPD, mean ± SD (%)	0.53 ± 0.23	0.52 ± 0.19	0.98
FD, mean ± SD	1.46 ± 0.15	1.43 ± 0.10	0.61
LAC, mean ± SD	2.10 ± 0.49	2.53 ± 1.26	0.33

Continuous variables are presented as mean ± standard deviation (SD). PCV = polypoidal choroidal vasculopathy. CNV = choroidal neovascularization. VPD = vascular perfusion density. FD = fractal dimension. LAC = lacunarity.

## Data Availability

Not applicable.
